# Effectiveness of 13-valent pneumococcal conjugate vaccine on radiological primary end-point pneumonia among cases of severe community acquired pneumonia in children: A prospective multi-site hospital-based test-negative study in Northern India

**DOI:** 10.1371/journal.pone.0276911

**Published:** 2022-12-15

**Authors:** Shally Awasthi, Neera Kohli, Monika Agarwal, Chandra Mani Pandey, Tuhina Rastogi, Anuj Kumar Pandey, Chittaranjan Roy, Kripanath Mishra, Neelam Verma, Chandra Bhushan Kumar, Pankaj Kumar Jain, Rajesh Yadav, Puneet Dhasmana, Abhishek Chauhan, Namita Mohindra, Ram Chandra Shukla

**Affiliations:** 1 Department of Pediatrics, King George’s Medical University, Lucknow, India; 2 Department of Radio-diagnosis, King George’s Medical University, Lucknow, India; 3 Department of Community Medicine, King George’s Medical University, Lucknow, India; 4 Department of Biostatistics and Health Informatics, Sanjay Gandhi Postgraduate Institute of Medical Sciences, Lucknow, India; 5 Department of Community Medicine, Darbhanga Medical College and Hospital, Darbhanga, India; 6 Department of Pediatrics, Darbhanga Medical College and Hospital, Darbhanga, India; 7 Department of Pediatrics, Patna Medical College and Hospital, Patna, India; 8 Department of Community Medicine, Uttar Pradesh University of Medical Sciences, Etawah, India; 9 Department of Pediatrics, Uttar Pradesh University of Medical Sciences, Etawah, India; 10 Department of Radio-diagnosis, Dr Ram Manohar Lohia Institute of Medical Sciences, Lucknow, India; 11 Department of Radio-diagnosis, Sanjay Gandhi Post Graduate Institute of Medical Sciences, Lucknow, India; 12 Department of Radio-diagnosis, Institute of Medical Sciences, Banaras Hindu University, Varanasi, India; University of Gondar, ETHIOPIA

## Abstract

**Introduction:**

Community acquired pneumonia (CAP) is a leading cause of under-five mortality in India and *Streptococcus pneumoniae* is the main bacterial pathogen for it. Pneumococcal Conjugate Vaccine 13 (PCV13) has been introduced in a phased manner, in the national immunization program of India since 2017/2018. The primary objective of this study was to evaluate the effectiveness of PCV13 on chest radiograph (CXR)-confirmed pneumonia, in children hospitalized with WHO-defined severe CAP.

**Methods:**

This prospective, multi-site test-negative study was conducted in a hospital-network situated in three districts of Northern India where PCV13 had been introduced. Children aged 2–23 months, hospitalized with severe CAP and with interpretable CXR were included after parental consent. Clinical data was extracted from hospital records. CXRs were interpreted by a panel of three independent blinded trained radiologists. Exposure to PCV13 was defined as ≥2 doses of PCV13 in children aged ≤ 12 months and ≥ 1 dose(s) in children > 12 months of age. Our outcome measures were CXR finding of primary endpoint pneumonia with or without other infiltrates (PEP±OI); vaccine effectiveness (VE) and hospital mortality.

**Results:**

From 1^st^ June 2017-30^th^ April 2021, among 2711 children included, 678 (25.0%) were exposed to PCV1. CXR positive for PEP±OI on CXR was found in 579 (21.4%), of which 103 (17.8%) were exposed to PCV. Adjusted odds ratio (AOR) for PEP±OI among the exposed group was 0.69 (95% CI, 0.54–0.89, p = 0.004). Adjusted VE was 31.0% (95% CI: 11.0–44.0) for PEP±OI. AOR for hospital mortality with PEP±OI was 2.65 (95% CI: 1.27–5.53, p = 0.01).

**Conclusion:**

In severe CAP, children exposed to PCV13 had significantly reduced odds of having PEP±OI. Since PEP±OI had increased odds of hospital mortality due to CAP, countrywide coverage with PCV13 is an essential priority.

## Introduction

Community acquired pneumonia (CAP) is a leading cause of potentially vaccine-preventable illness and death among under-five children in India. Globally, the number of deaths due to pneumonia among under-five children was 0·8 million in 2018 [1]. Nigeria had the highest number of deaths followed by India (0.13 million), Pakistan, the Democratic Republic of Congo and Ethiopia. These five countries combined account for more than half of total pneumonia deaths among the under-five children [1].

Pneumococcal conjugate vaccine (PCV) has been recommended by the World Health Organization (WHO) to prevent CAP [2]. WHO recommends its inclusion in the national immunization program (NIP) of countries with high CAP related morbidity and mortality [2,3]. In compliance with the WHO recommendations, the Government of India, introduced PCV13 (Prevnar ® by Pfizer) in the NIP on 1^st^ June of 2017 onwards in a phased manner at select states (Bihar, Himachal Pradesh, Madhya Pradesh, Rajasthan and Uttar Pradesh) and within states select districts [4]. The dose-schedule of PCV13 in NIP of India is 6 weeks, 14 weeks and 9 months (booster dose) [4,5]. A reduction of CAP among children, after the introduction of PCV13 has been reported in different settings [6,7].

We analyzed data from an ongoing multi-site study to evaluate the effectiveness of PCV13 on chest radiograph (CXR)-confirmed pneumonia, in children hospitalized with WHO-defined severe CAP in three districts of Northern India [8]. We also compared hospital mortality among those with primary endpoint pneumonia with or without other infiltrates (PEP±OI) on CXR as a secondary objective. This work was done as part of a hospital-based surveillance on CAP among children (2–59 months) ongoing in these three districts in India since 1^st^ January, 2015 [8].

## Methods

The study analyzed data from three districts of Uttar Pradesh and Bihar states of Northern India. Uttar Pradesh is the fourth largest state by area (93,023 mi^2^) and is the most populous in India [9]. Bihar has an area of 36,357 mi^2^ and is the third most populous state [10]. This analysis reports data of Darbhnaga district of Bihar where PCV13 was introduced on 1^st^ June, 2017 and data from Lucknow (Uttar Pradesh) and Patna (Bihar) districts where PCV13 was introduced on 1^st^ June, 2018.

A `test-negative`study design was used in this analysis to estimate the vaccine effectiveness (VE) [11]. We used the same clinical case definition to enroll both the cases and controls [11]. However, the cases and controls differed with respect to their CXR findings [11]. A child was considered `test-positive`if the CXR finding was PEP± OI and `test -negative`if findings on CXR were either `normal`or `other infiltrates only`.

An active, hospital-based surveillance network was established for this study [8]. Hospitals included in the analysis were private and public hospitals that admitted pediatric patients. A total of 92 public and private hospitals participated from three districts whose data was analyzed. Recruitment was done by trained surveillance officers [8,12,13]. From 1^st^ June 2017/2018-30^st^ April 2021, surveillance officers identified children from each participating hospital by reviewing admission logbooks. Children of eligible age (2–59 months), admitted with history of fast breathing with chest in-drawing were identified from hospital records. Children were included if they were hospitalized with symptoms of WHO-defined severe CAP, were permanent resident of the project district, had illness of <14 days and were neither hospitalized nor recruited previously in the study. Excluded were those with cough for ≥14 days or prior hospitalization. Excluded from the analysis were children ≥ 24 months of age as they were not eligible for PCV13. `*Pneumonia*`was defined as fast breathing above age-specific cut-off (≥50 breaths/min between 2–11 months and ≥40 breaths/min between 12–59 months) with/without cough/fever [14]. Child was classified as having `*severe pneumonia*`in the presence of at least one of the following: (a) oxygen saturation <90% or central cyanosis or (b) severe respiratory distress (e.g., grunting, very severe chest in-drawing) or (c) signs of pneumonia with a general danger sign (inability to breast feed or drink, lethargy or reduced level of consciousness, convulsions) or (d) severe malnutrition [14].

After obtaining written, informed consent from the parents/legal guardians, trained surveillance officers interviewed them to obtain socio-demographic information. Information on age, gender, place of residence, family type, breastfeeding status and smoking status of the parents was noted. Family type was categorized into nuclear and joint. A family was considered nuclear if the family had a nuclear pair comprising of head and spouse with or without unmarried children [15]. A family that was not nuclear was considered joint [15]. Anthropometry (weight and height) were noted from the hospital records. Standardized questionnaire has been published elsewhere [12].

PCV13 status was noted from the vaccination card issued by the immunization clinic and available with the parents at the time of data collection. If the card was unavailable, the parents/caregivers were requested to provide the digital image of the card to the surveillance officer or the surveillance officer asked for the date(s) of vaccination by calling a family member at home who read it out from the vaccination card. A child was considered un-immunized in case the parents/caregivers were unable to provide the information about vaccination. Children ≤ 12 months of age who received ≥2 doses of PCV13 and children > 12 months of age who received at least one dose of PCV13 were considered `*exposed to PCV`*, otherwise `*non-exposed to PCV*`.

Clinical data was recorded by pre-existing trained hospital staff at the time of hospitalization. They noted information for the following variables: respiratory rate, heart rate, axillary temperature ≥37.5°C (yes or no with duration if yes), pallor, presence of any general danger sign (inability to breast feed or drink, lethargy or reduced level of consciousness, convulsions, vomiting everything, grunting, severe malnutrition), oxygen saturation by pulse oximetry and central cyanosis. Findings on auscultation of chest was noted by the treating physician. The data were extracted from the hospital records by project staff. Clinical outcome (discharge or death) was noted from the hospital logbook on follow-up. Detailed methodology of data collection has been published elsewhere [8,12,13].

CXRs were done on advice of the treating physician and a copy of the CXR was collected from the hospital by the surveillance officer. These CXRs were either analogue or digital. All CXRs were digitalized and stored online at www.capxrs.org. A panel of trained radiologists, using WHO methodology, interpreted CXRs. They first categorized the quality of film as interpretable or un-interpretable. Interpretable CXRs were classified as either `*optimal/adequate*`or `s*uboptimal*”. Thereafter, CXRs were interpreted for radiological abnormality, and categorized as abnormal or normal. An abnormal CXR was categorized as ‘PEP only’ or ‘other infiltrates only’ or ‘both PEP and other infiltrates`[16]. Outcome of interest was the presence of PEP±OI on CXR and these were `*cases*`. The rest were categorized as `*controls*`. Standardized WHO case definition of PEP ±OI has been used [16] and has been reported elsewhere [12].

### Definitions

Dependent variable was case-control status. Cases were those who had PEP±OI on CXR and controls were those who had either normal CXRs or only OI. Weight-for-age (WAZ) and height-for-age (HAZ) z-score of each child was calculated using WHO Anthro Survey Analyzer [17]. Malnutrition status were categorized as WAZ >-2SD (normal), WAZ ≤ -2SD (malnourished) and WAZ ≤ -3SD (severe malnutrition) [17]. Hypoxia was defined as oxygen saturation<90% on pulse oximetry or requiring oxygen supplementation during hospital stay [18,19]. Since respiratory syncytial virus (RSV) tends to peak in India in five months of early winter (June to October) and three months of winter (December to February), therefore these eight months were considered as RSV season [20].

### Sample size

About 25% children had PEP±OI on CXR among the total children recruited in the current surveillance^13^. We assume that there would be a 25% reduction [21] in the occurrence of PEP±OI, in children `*exposed to PCV*`. For an α = 0.05 level of significance and power of 90%, and 1:3 case to controls, we required a minimum sample size of 475 in `exposed to PCV`and 1423 in `non-exposed to PCV`group. Assuming non-response rate of 10%, the required sample size was 522 in exposed and 1565 in non-exposed groups, with total sample size of 2087.

### Statistical analysis

Overall descriptive statistics of independent variables, such as socio-demographic and clinical were imputed. Weight of 7.2% (196/2711) and height of 9.4% (255/2711) children were missing in our data. Missing weight and height were estimated using regression-based imputation technique [22]. Number (percentage) is being reported for categorical data. Mean and standard deviation (S.D.) or median with interquartile range (IQR) for continuous data was calculated and reported. Statistical Package of Social Science (SPSS version 24) [23] software was used to perform statistical analysis.

We compared independent variables among cases and control and also among those who were *`exposed*`and *`non-exposed*`to PCV. We used chi-square test for comparison of categorical data and student’s t-test for normal continuous data. Mann-Whitney U test was used for non-normal data. A p value of < 0·05 was taken as statistically significant using a two-tailed distribution.

A directed acyclic graph (DAG) approach was used to increase precision of the analysis [24]. Variables included in DAG were clustered in four different groups: (i) *Exposure variable*: Vaccination with PCV13 (ii) *Outcome variable*: PEP±OI (iii) *Potential confounder variables*: child`s age (in months), gender, birth order, immunization other than PCV, nutritional status (malnutrition), education status of parents, seasonality, resident district and year of enrollment (iv) *Mediator variables*: hypoxia, duration of breastfeeding ≥ 6 months or currently breastfed, any co-morbidity, use of biomass fuel for cooking. We used potential confounders in the logistic regression model as these were found to be associated with exposure to PCV.

Independent variables that had a univariate association with two-tailed p value ≤ 0·1 with outcome or were listed as potential confounders in the DAG were used in the regression model. Logistic regression was done where dependent variable was CXR abnormality (PEP±OI versus others). Unadjusted odds ratio (OR) for univariate comparison and adjusted OR (AOR) for multivariate comparison with 95% confidence interval (CI) are being reported. Also, VE against PEP ± OI was calculated by using the formula VE = (1-OR) ×100% [20].

We compared independent variables among children of CAP with and without hospital mortality and predicted hospital mortality by using step-wise logistic regression model. We calculated AOR for hospital mortality with 95% CI. Independent variable that had a univariate association with hospital mortality with a two-tailed p value ≤ 0.1 were used in logistic regression model. Unadjusted OR is also being reported.

### Patient and public involvement

Patients and the public were not involved in the design, conduct, analysis or interpretation of the study.

### Ethical approval

The study was ethically reviewed and approved by the Ethics Review Committee of each site. Details of ethical approval are as follows: (i) King George`s Medical University, Lucknow vide letter no. 2800 Ethics/R Cell-14 dated 22nd November, 2014 (ii) Darbhanga Medical College & Hospital, Darbhanga vide letter no. 05/IEC/DMC dated 19th February, 2015 (iii) Patna Medical College and Hospital, Patna vide letter no. nil dated 15th October, 2015. The caregivers/guardians of children signed the written, informed consent for participation in study.

## Results

**Fig 1:**
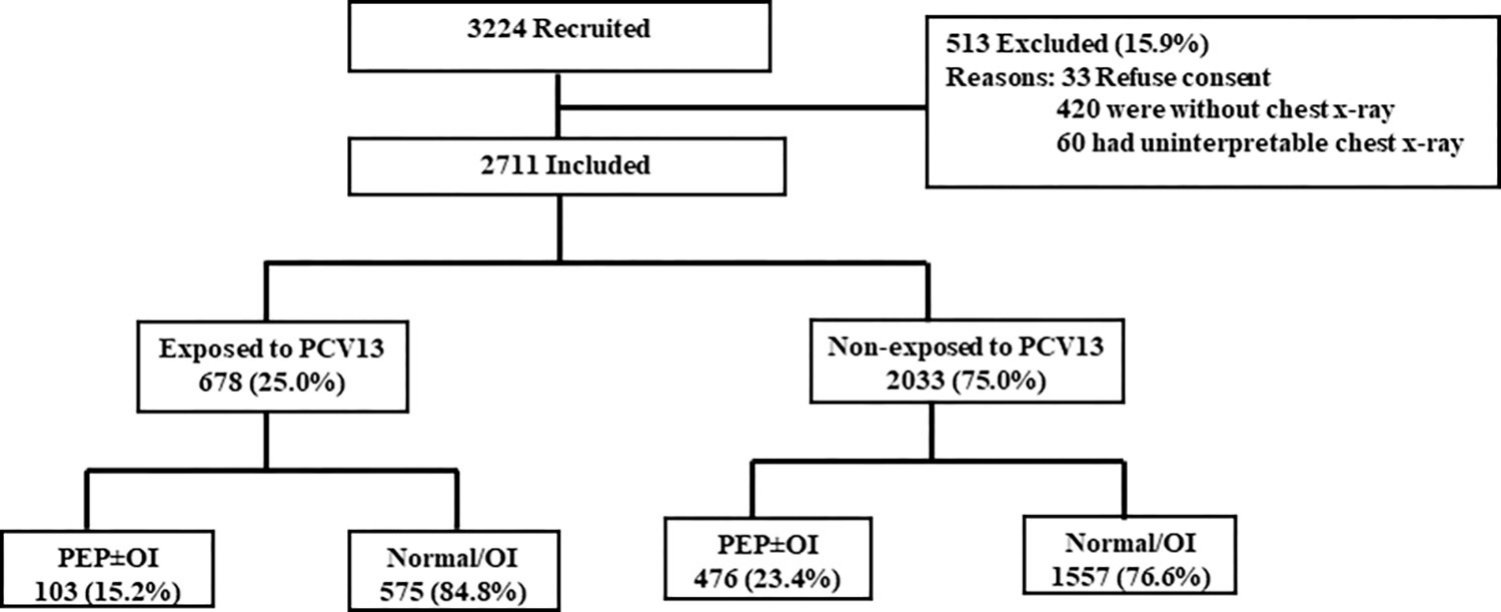
Flow diagram of hospitalized children (2–23 months) with community acquired pneumonia recruited from Darbhanga (1^st^ June 2017-30^th^ April 2021), Patna (1^st^ June 2018 to 30^th^ April 2021) and Lucknow (1^st^ June 2018 to 30^th^ April 2021).

This analysis was done as part of a hospital-based surveillance on CAP among children (2–23 months) on data collected between 1^st^ June, 2017-30th April, 2021 at Darbhanga district and between 1^st^ June, 2018-30th April, 2021 at Patna and Lucknow districts respectively. A total of 3224 children, hospitalized with severe CAP, met the eligibility criteria. Among the eligible children, 15.9% (513/3224) were excluded. Details of excluded children are given in Fig 1. Thereafter, 2711 children were included for the analysis.

Among the 2711 included children, 25.0% (678/2711) were `*exposed to PCV*`. Of these 15.2% (103/678) had PEP±OI (test-positive), and 84.8% (575/678) had normal CXRs or other infiltrates (test-negative). Among 75.0% (2033/2711) children that were `*non-exposed to PCV*`, 23.4% (476/2033) had PEP±OI, and 76.6% (1557/2033) had normal CXRs or other infiltrates (Fig 1).

The median age of children with CAP (n = 2711) was 6 months (IQR: 3–10) and among these 79.2% (2147/2711) were <12 months of age. Females were lesser in proportion (30.0%, 813/2711) compared to males. There was a district-wide variation among children who had PEP±OI on CXR. A significant difference was observed in socio-demographic characteristics notably in age (months), gender (female), family type, other immunization status (excluding PCV), wheezing, HAZ, WAZ, and hypoxia among those with or without PEP±OI (Table 1). In clinical features, except cough, all the variables were similar among children with or without PEP±OI. Among the general danger signs, all the variables were significantly different except inability to drink and lethargy/unconscious by PEP±OI (Table 1).

**Table 1:** Overall descriptive statistics and comparison of sociodemographic and clinical variables among the children with or without PEP±OI.

Characteristics	Overall	PEP±OI	Normal/OI	
Sociodemographic characteristics	N = 2711 (%)	N = 579 (%)	N = 2132 (%)	p value
Age (in months) median (IQR)	6 (3–10)	5 (3–10)	6 (4–11)	<0.001
Age 2–11 months n (%)	2147 (79.2)	471 (81.3)	1676 (78.6)	0.15
Weight for age, mean (SD)	-1.51 (1.60)	-1.79 (1.66)	-1.43 (1.58)	<0.001
Height for age, mean (SD)	-1.84 (1.77)	-2.03 (1.74)	-1.79 (1.77)	<0.001
Gender (female) n (%)	813 (30.0)	201 (34.7)	612 (28.7)	0.005
Residence (rural) n (%)	1579 (58.2)	338 (58.4)	1241 (58.2)	0.94
^a^Season (RSV) n (%)	1886 (69.6)	420 (72.5)	1466 (68.8)	0.08
Biomass fuel n (%)	1247 (46.0)	257 (44.4)	990 (46.4)	0.38
Pallor n (%)	1387 (51.2)	302 (52.2)	1085 (50.9)	0.59
Wheezing n (%)	2136 (78.8)	429 (74.1)	1707 (80.1)	0.002
Hypoxia n (%)	1559 (57.5)	354 (61.1)	1205 (56.5)	0.046
^c^Co-morbidities n (%)	101 (3.7)	24 (4.1)	77 (3.6)	0.55
^b^Breastfeed n (%)	2452 (90.4)	515 (88.9)	1937 (90.9)	0.17
Family type (Joint) n (%)	1914 (70.6)	428 (73.9)	1486 (69.7)	0.048
**Mother’s education (N = 2687, row %)**				
No formal education	1094 (100.0)	233 (21.3)	861 (78.7)	0.93
Formal education	1593 (100.0)	337 (21.2)	1256 (78.8)	
**Father’s education (N = 2687, row %)**				
No formal education	872 (100.0)	200 (22.9)	672 (77.1)	0.13
Formal education	1815 (100.0)	370 (20.4)	1445 (79.6)	
**Father’s smoking (N = 2683, row %)**				
Yes	434 (100.0)	106 (24.4)	328 (75.6)	0.07
No	2249 (100.0)	462 (20.5)	1787 (79.5)	
**Participating sites n (%)**				
Lucknow	1007 (37.1)	242 (41.8)	765 (35.9)	<0.001
Patna	555 (20.5)	145 (25.0)	410 (19.2)	
Darbhanga	1149 (42.4)	192 (33.2)	957 (44.9)	
**Type of house n (%)**				
Mud	549 (20.3)	129 (22.3)	420 (19.7)	0.13
Bricks	1559 (57.5)	337 (58.2)	1222 (57.3)	
Semi-constructed	603 (22.2)	113 (19.5)	490 (23.0)	
^**d**^ **Year of enrollment n (%)**				
2017	319 (11.8)	56 (9.7)	263 (12.3)	0.13
2018	845 (31.2)	176 (30.4)	669 (31.4)	
2019	1109 (40.9)	252 (43.5)	857 (40.2)	
2020	338 (12.5)	67 (11.6)	271 (12.7)	
2021	100 (3.7)	28 (4.8)	72 (3.4)	
**Birth order (N = 2689, row %)**				
First	984 (100.0)	212 (37.1)	772 (78.5)	0.82
Second	918 (100.0)	187 (20.4)	731 (79.6)	
Third	485 (100.0)	103 (21.2)	382 (78.8)	
More than third	302 (100.0)	69 (22.8)	233 (77.2)	
**Other immunization n (%)**				
Complete for age	2130 (78.6)	427 (73.7)	1703 (79.9)	0.001
Incomplete/unimmunized	581 (21.4)	152 (26.3)	429 (20.1)	
**Clinical features**				
Fever	2341 (86.4)	504 (87.0)	1837 (86.2)	0.58
Cough	2684 (99.0)	569 (98.3)	2115 (99.2)	0.046
Fast breathing	2365 (87.2)	515 (88.9)	1850 (86.8)	0.17
Difficult breathing	2687 (99.1)	573 (99.0)	2114 (99.2)	0.66
**General danger sign**				
Inability to drink	1386 (51.1)	299 (51.6)	1087 (51.0)	0.78
Vomiting everything	1027 (37.9)	185 (32.0)	842 (39.5)	0.001
Convulsion	243 (9.0)	37 (6.4)	206 (9.7)	0.02
Lethargy/unconscious	1576 (58.1)	338 (58.4)	1238 (58.1)	0.89
Grunting	2141 (79.0)	438 (75.6)	1703 (79.9)	0.03
Severe malnutrition	454 (16.7)	137 (23.7)	317 (14.9)	<0.001
^**e**^ **PCV13 immunity n (%)**				
Exposed to PCV	678 (25.0)	103 (17.8)	575 (27.0)	<0.001
Non-exposed	2033 (75.0)	476 (82.2)	1557 (73.0)	

^a^*Season*: *RSV season refers to period between June to October and December to February*.

^b^*Breastfeed*: *Duration of breastfeed ≥ 6 months or currently breastfeed*.

^d^*Year of enrollment refers to children enroll from 1*^*st*^ *June 2017 to 30*^*th*^ *April 2021*.

^c^*Co-morbidities*: *Congenital heart disease and history of fast breathing and cough ≥ 3 times in 6 months*.

^e^*PCV13 immunity*: *Children ≤ 12 months of age who received >2 PCV doses and children > 12 months of age who received at least one dose of PCV were considered `exposed to PCV`*, *else `non-exposed to PCV`*.

We compared the socio-demographic and clinical variables among children that were or were not exposed to PCV. Significant differences were observed between those `*exposed*`and `*non-exposed to PCV*`in the socio-demographic and clinical variables. Younger children were more `*non-exposed to PCV*`compared to `*exposed to PCV*`(80.2% vs 76.3%). Children who were `*non-exposed to PCV*`were more likely to be females, living in rural areas, parents had no formal education, family used biomass fuel for cooking, had either incomplete immunization (excluding PCV) or were unimmunized, were hypoxic on hospitalization, had any co-morbidity or were severely malnourished as compared to the `*exposed to PCV`*group. There was a significant difference observed between *`exposed*`and `*non-exposed*`to PCV groups with respect to participating sites, house type, year of enrollment and CXR abnormalities (Table 2).

**Table 2:** Comparison of sociodemographic and clinical variables among the children exposed or unexposed to PCV13 vaccination.

Characteristics	Exposed PCV13	Non-exposed PCV13	
Sociodemographic characteristics	N = 678 (%)	N = 2033 (%)	p value
Age 2–11 months n (%)	517 (76.3)	1630 (80.2)	0.03
Gender (female) n (%)	182 (26.8)	631 (31.0)	0.04
Residence (rural) n (%)	369 (54.4)	1210 (59.5)	0.02
Season (RSV) n (%)	465 (68.6)	1421 (69.9)	0.52
Breastfeed n (%)	606 (89.4)	1846 (90.8)	0.28
Family type (Joint)^c^ n (%)	471 (69.5)	1443 (71.0)	0.46
Biomass fuel n (%)	257 (37.9)	990 (48.7)	<0.001
Pallor n (%)	355 (52.4)	1032 (50.8)	0.47
Wheezing n (%)	554 (81.7)	1582 (77.8)	0.03
Hypoxia^a^ n (%)	360 (53.1)	1199 (59.0)	0.007
Co-morbidities^b^ n (%)	15 (2.2)	86 (4.2)	0.02
Severe malnutrition n (%)	76 (11.2)	378 (18.6)	<0.001
**Mother’s education (Row %)**			
No formal education (n = 1094)	210 (19.2)	884 (80.8)	<0.001
Formal education (n = 1593)	464 (29.1)	1129 (70.9)	
**Father’s education (Row %)**			
No formal education (n = 872)	166 (19.0)	706 (81.0)	<0.001
Formal education (n = 1815)	507 (27.9)	1308 (72.1)	
**Father’s smoking (Row %)**			
Yes (n = 434)	94 (21.7)	340 (78.3)	0.07
No (n = 2249)	579 (25.7)	1670 (74.3)	
**Participating sites**			
Lucknow	299 (44.1)	708 (34.8)	<0.001
Patna	51 (7.5)	504 (24.8)	
Darbhanga	328 (48.4)	821 (40.4)	
**Type of house n (%)**			
Mud	100 (14.7)	449 (22.1)	<0.001
Bricks	426 (62.8)	1133 (55.7)	
Semi-constructed	152 (22.4)	451 (22.2)	
**Birth order (Row %)**			
First (n = 984)	265 (26.9)	719 (73.1)	0.21
Second (n = 918)	228 (24.8)	690 (75.2)	
Third (n = 485)	106 (21.9)	379 (78.1)	
More than third (n = 302)	75 (24.8)	227 (75.2)	
**Year of enrollment n (%)**			
2017	15 (2.2)	304 (15.0)	<0.001
2018	123 (18.1)	722 (35.5)	
2019	364 (53.7)	745 (36.6)	
2020	143 (21.1)	195 (9.6)	
2021	33 (4.9)	67 (3.3)	
**Other immunization n (%)**			
Complete for age	636 (93.8)	1494 (73.5)	<0.001
Incomplete/unimmunized	42 (6.2)	539 (26.5)	
**Chest x-rays findings n (%)**			
PEP with or without infiltrates	103 (15.2)	476 (23.4)	<0.001
Normal/other infiltrates	575 (84.8)	1557 (76.6)	

^a^Hypoxia: Oxygen saturation<90% on pulse oximetry or requiring oxygen supplementation during hospital stay.

^b^Co-morbidities: Congenital heart disease and history of fast breathing and cough ≥ 3 times in 6 months.

^c^Joint: A family that was not nuclear.

In bivariable model, unadjusted OR for `*exposed to PCV*`group against PEP±OI was 0.59 (95% CI 0.46–0.74) and unadjusted VE was 41.0% (95% CI, 26.0–54.0). In multivariable model, AOR was estimated by using step-wise logistic regression method, adjusted for age (in months), parent’s education, RSV season, gender, other immunization (excluding PCV), malnutrition, birth order, year of enrollment, and participating sites. We have not used co-morbidity in the model as there were collinearity with malnutrition (Unadjusted OR 2.11, 95% CI;1.29–3.44, p value = 0.003). We found that children `*exposed to PCV*`had reduced the odds of having PEP±OI (AOR 0.69, 95% CI, 0.54–0.89). Adjusted VE was 31.0% (95% CI, 11.0–44.0) for PEP±OI (Table 3).

**Table 3:** Association of chest x-ray abnormalities with PCV13, controlling for independent variables.

Variables	Model PEP with or without other infiltrates/Normal & OI^ref^			
	Bivariable		Multivariable	
	Unadjusted OR (95% CI)	p value	Adjusted OR (95% CI)	p value
Age (in months) (2–11)	1.19 (0.94–1.50)	0.15		
Mother’s education (without formal)	1.01 (0.84–1.22)	0.93		
Season (RSV)	1.20 (0.98–1.46)	0.08		
Exposed PCV13	0.59 (0.46–0.74)	**<0.001**	0.69 (0.54–0.89)	**0.004**
Gender (female)	1.32 (1.09–1.61)	**0.005**	1.24 (1.02–1.52)	**0.04**
Father’s education (without formal)	1.16 (0.96–1.41)	0.13	1.22 (0.99–1.51)	**0.07**
Other immunization (Incomplete for age)	1.41 (1.14–1.75)	**0.001**	1.23 (0.99–1.54)	**0.07**
Malnutrition status (Severe malnutrition)	1.83 (1.45–2.31)	**<0.001**	1.72 (1.35–2.20)	**<0.001**
**Birth Order**				
First	Reference	-		
Second	0.93 (0.75–1.16)	0.53		
Third	0.98 (0.75–1.28)	0.89		
More than third	1.08 (0.79–1.47)	0.63		
**Year of enrollment**				
2017	Reference	-		
2018	1.24 (0.89–1.72)	0.21		
2019	1.38 (1.00–1.90)	**0.049**		
2020	1.16 (0.78–1.72)	0.46		
2021	1.83 (1.08–3.08)	**0.02**		
**Districts**				
Darbhanga	Reference	-	Reference	-
Lucknow	1.58 (1.28–1.95)	**<0.001**	1.70 (1.35–2.14)	**<0.001**
Patna	1.76 (1.38–2.25)	**<0.001**	1.65 (1.28–2.12)	**<0.001**

**Abbreviations:** PEP; Primary endpoint pneumonia; PCV13: Pneumococcal conjugate vaccine13; CXR: Chest x-ray; OI: Other infiltrates; OR: Odds ratio; CI: Confidence interval.

We compared the sociodemographic and clinical variables among cases of CAP with or without hospital mortality. Mortality of hospitalized child was associated with parents having no formal education (Table 4). Children having hypoxic pneumonia, any co-morbidity, severe malnutrition, and CXR abnormalities (PEP±OI) had higher odds of hospital mortality (Table 4). Unadjusted OR of hospital mortality among children with PEP±OI on CXR was 2.91 (95% CI, 1.44–5.89, p = 0.003) while the AOR of hospital mortality was 2.65 (95% CI, 1.25–5.53, p = 0.01).

**Table 4:** Comparison of sociodemographic and clinical variables among the cases of CAP with and without hospital mortality.

Characteristics	Dead	Alive	
Sociodemographic characteristics	N = 32 (%)	N = 2679 (%)	p value
Age 2–11 months n (%)	25 (78.1)	2122 (79.2)	0.88
Gender (female) n (%)	13 (40.6)	800 (29.9)	0.19
Residence (rural) n (%)	21 (65.6)	1558 (58.2)	0.39
Family type (Joint) n (%)	23 (71.9)	1891 (70.6)	0.87
Season (RSV) n (%)	22 (68.8)	1864 (69.6)	0.92
Breastfeed n (%)	29 (90.6)	2423 (90.4)	0.97
Biomass fuel n (%)	19 (59.4)	1228 (45.8)	0.13
Pallor n (%)	12 (37.5)	1375 (51.3)	0.12
Wheezing n (%)	24 (75.0)	2112 (78.8)	0.60
Hypoxia n (%)	31 (96.9)	1528 (57.0)	**<0.001**
Co-morbidities^a^ n (%)	9 (28.1)	92 (3.4)	**<0.001**
Severe malnutrition n (%)	14 (43.8)	440 (16.4)	**<0.001**
**Mother’s education (Row %)**			
No formal education (n = 1094)	19 (1.7)	1075 (98.3)	**0.03**
Formal education (n = 1593)	13 (0.8)	1580 (99.2)	
**Father’s education (Row %)**			
No formal education (n = 872)	17 (1.9)	855 (98.1)	**0.01**
Formal education (n = 1815)	15 (0.8)	1800 (99.2)	
**Father’s smoking (Row %)**			
Yes (n = 434)	8 (1.8)	426 (98.2)	0.17
No (n = 2249)	24 (1.1)	2225 (98.9)	
**Participating sites**			
Lucknow	9 (28.1)	998 (37.3)	0.55
Patna	8 (25.0)	547 (20.4)	
Darbhanga	15 (46.9)	1134 (42.3)	
**Type of house n (%)**			
Mud	7 (21.9)	542 (20.2)	0.97
Bricks	18 (56.3)	1541 (57.5)	
Semi-constructed	7 (21.9)	596 (22.2)	
**Birth order (Row %)**			
First (n = 984)	11 (1.1)	973 (98.9)	0.46
Second (n = 918)	13 (1.4)	905 (98.6)	
Third (n = 485)	7 (1.4)	478 (98.6)	
More than third (n = 302)	1 (0.3)	301 (99.7)	
**Year of enrollment n (%)**			
2017	5 (15.6)	314 (11.7)	0.38
2018	8 (25.0)	837 (31.2)	
2019	17 (53.1)	1092 (40.8)	
2020	2 (6.3)	336 (12.5)	
2021	0 (0.0)	100 (3.7)	
**Other immunization n (%)**			
Complete for age	22 (68.8)	2108 (78.7)	0.17
Incomplete/unimmunized	10 (31.3)	571 (21.3)	
**PCV13 status**			
Exposed to PCV13	4 (12.5)	674 (25.2)	0.10
Non-exposed to PCV13	28 (87.5)	2005 (74.8)	
**Chest x-rays findings n (%)**			
PEP with or without infiltrates	14 (43.8)	565 (21.1)	**0.002**
Normal/other infiltrates	18 (56.3)	2114 (78.9)	

^a^*Co-morbidities*: *Congenital heart disease and history of fast breathing and cough ≥ 3 times in 6 months*.

## Discussion

In this prospective, multi-site, test negative study, we found that exposure to PCV13 had significantly reduced the odds of having PEP±OI on CXRs. Adjusted VE for PEP±OI was found to be 31.0% (95% CI, 11.0–44.0). CXRs were evaluated by a panel of trained external radiologists using standard WHO-methodology of CXR interpretation [13]. The study therefore had internal as well and external validity and can be compared to studies that used similar methodology [25,26].

The single, largest infectious cause of death among children worldwide is pneumonia, caused by viruses, bacteria and fungi [27]. *Streptococcus pneumoniae* (SP) is the most common bacterial cause of CAP in children [27]. However, there is a dearth of data on invasive bacterial pathogens, isolated by culture of sterile body fluids, causing pneumonia. Etiological association between microorganism(s) and pneumonia has been assessed by molecular techniques over the last one decade. A case-control, multi-site study (including India as a site) reported that adjusted population attributable fraction for association of SP with pneumonia was 42.2% (95 CI: 35.5%-48.2%), followed by respiratory syncytial virus or RSV (18.2%; 95% CI: 17.4%-19.0%), and rhinovirus (11.2%; 95% CI, 7.5%-14.7%) [28]. Similar findings were reported by primarily single-center studies conducted in India [29]. A case-control study entitled Pneumonia Aetiology Research for Child Health (PERCH) investigated the etiology for CAP using molecular as well as culture methods [30]. PERCH, a multi-centric study conducted in seven countries (Gambia, Kenya, Mali, South Africa, Zambia, Thailand, and Bangladesh), RSV as the most common cause, specifically for children <6 months of age [30]. Bacterial pathogens, including SP, caused substantial proportion of fatality among children, which can be prevented by early access to treatment and increased coverage with PCV13 [29]. This also makes a case for early and widespread use of PCV13 vaccine against SP [29].

In our study, among the 2711 included children, 25.0% (678/2711) were `*exposed to PCV13*`. This happened because the Government of India introduced PCV13 in a phased manner across the country from 1^st^ June in 2017 onwards and even within allotted districts the vaccine coverage was taking time to optimize [4]. A WHO-UNICEF Survey has reported that in 2018 and 2019 only 44% and 57%, of the surviving infants from the target population in India, had received all the three doses of PCV13 [31].

Effectiveness of PCV vaccine to reduce radiological pneumonia among young children from other countries has been widely reported in scientific literature [7,17,32]. A South African trial reported that PCV9 reduced the incidence of first episodes of radiologically confirmed alveolar consolidation by almost one fourth in children without human immunodeficiency virus (HIV) infection [32]. This is slightly lower than 31% odds of reduction in PEP±OI in our study. Since our study area has a low prevalence of HIV [33], subjects were not tested for it. A population-based surveillance in Gambia on children (2–59 months) reported about 24% reduction in the incidence of radiological pneumonia with consolidation in children aged 2–59 months following the introduction of PCV [17]. The same study reported VE increased with greater numbers of doses [17].

In our analysis, we found that presence of PEP±OI was associated with almost three times the odds of hospital mortality in severe CAP. In a trial of PCV9 in South Africa, there was an insignificant reduction in all-cause as well as pneumonia specific mortality among the HIV infected as well as uninfected children [32]. In contrast, a population-based case-control study from Chile reported VE estimates of PCV10 to be 71.5 on pneumonia deaths and 34.8 on all-cause deaths [34]. Similar findings were reported from a time-series analysis conducted in Peru against pneumonia-related mortality [35]. Hence, the introduction of PCV would reduce the proportion of PEP±OI in children with CAP and hospital mortality.

We observed that no formal education of parents, presence of hypoxic pneumonia, co-morbidities, severe malnutrition, and CXR abnormalities (PEP±OI) were significantly associated with hospital mortality. Our results are in concurrence with those by PERCH multi-centric study that also reported that those with consolidation on CXR were at significantly higher risk of death than those with a normal CXR or with other infiltrates only [36]. Hypoxic pneumonia is a known marker of severe pneumonia and a precursor to mortality [20]. Similar to our study, previous studies have established association of hypoxic pneumonia with mortality [20,37,38] and found that PCV13 is effective against the hypoxic pneumonia [20] and hence cause-specific [39,40] and all-cause mortality [39,40].

In the current study, etiology of CAP was not investigated. RSV is an important, perhaps the leading cause of CAP. Since RSV peaks between June to October months in India and shows smaller peak in December, January and February months [22], therefore RSV seasonality was controlled in our analysis. Also, the coverage of PCV13 is likely to increase with increase in number of years since its introduction. Increase in coverage is known to provide indirect protective benefit [41], hence the year of enrollment was a confounder in our analysis. In our study, there was a gradual increase in the number of enrolled children as between 1^st^ June, 2017 to 1^st^ June, 2018 only Darbhanga site was enrolling children; however between 1^st^ June 2018 to 30^th^ April, 2021 all three sites (Darbhanga, Lucknow and Patna) enrolled. Between 1^st^ March, 2020 to 30^th^ April, 2021 there was a dip in number of enrolled children from all sites due to COVID-19 pandemic. Gender disparity in health care-seeking for females is common in India [13], and in other South Asian countries [42]. Gender was therefore kept as a confounder in our analysis. Previous studies have reported that child`s age (<1 year) [43], maternal education [43,44], incomplete immunization/un-immunized [43,44], malnutrition [44,45] and resident district [46] were important risk factors of CAP in resource-deprived settings and were therefore confounders in our study.

### Strengths and limitations of the study

The study has several strengths. This analysis was done on data collected from a multi-site hospital-based surveillance for radiological pneumonia in which standardized definition of CAP was used for recruitment [14]. Uniform methodology of data captured by trained surveillance officers ensured quality. We also used WHO-recommended methodology for interpreting CXRs [16] which was done by a panel of trained radiologists [13], who had been a part of the study since its inception in 2015. This ensured internal and external validity. Use of test-negative design helped to reduce bias associated with confounding by health-care-seeking behavior [11]. DAG approach reduced bias, improved transparency and increased precision on our analysis [24]. Use of data of three representative sites and standardized methods will help in extrapolating our results to other similar sites in Northern India. The study had multiple hospitals in the surveillance network where uniform treatment protocols could not be followed. The different treatment protocols in such settings might have influenced the mortality and is hence a limitation. Also, longitudinal follow-up of the recruited children was not done. We also did not investigate for the etiological agents of CAP.

## Conclusion

Introduction of PCV vaccine has been found to reduce the proportion of cases with radiological pneumonia in previous [13] as well as current study. In our test-negative study design, VE of PEP ±OI was 31.0%. Since the PEP±OI was associated with increased odds of hospital mortality due to CAP, countrywide coverage with PCV13 is an essential priority.

## Supporting information

S1 Fig Google map of hospitals network in three districts of North India. (TIF)
