# Supplementary figures and images for: Effectiveness of 13-valent pneumococcal conjugate vaccine on radiological primary end-point pneumonia among cases of severe community acquired pneumonia in children: A prospective multi-site hospital-based test-negative study in Northern India
Source: PLoS One. 2022 Dec 15;17(12):e0276911. doi: 10.1371/journal.pone.0276911 (PMC9754232; doi:10.1371/journal.pone.0276911)

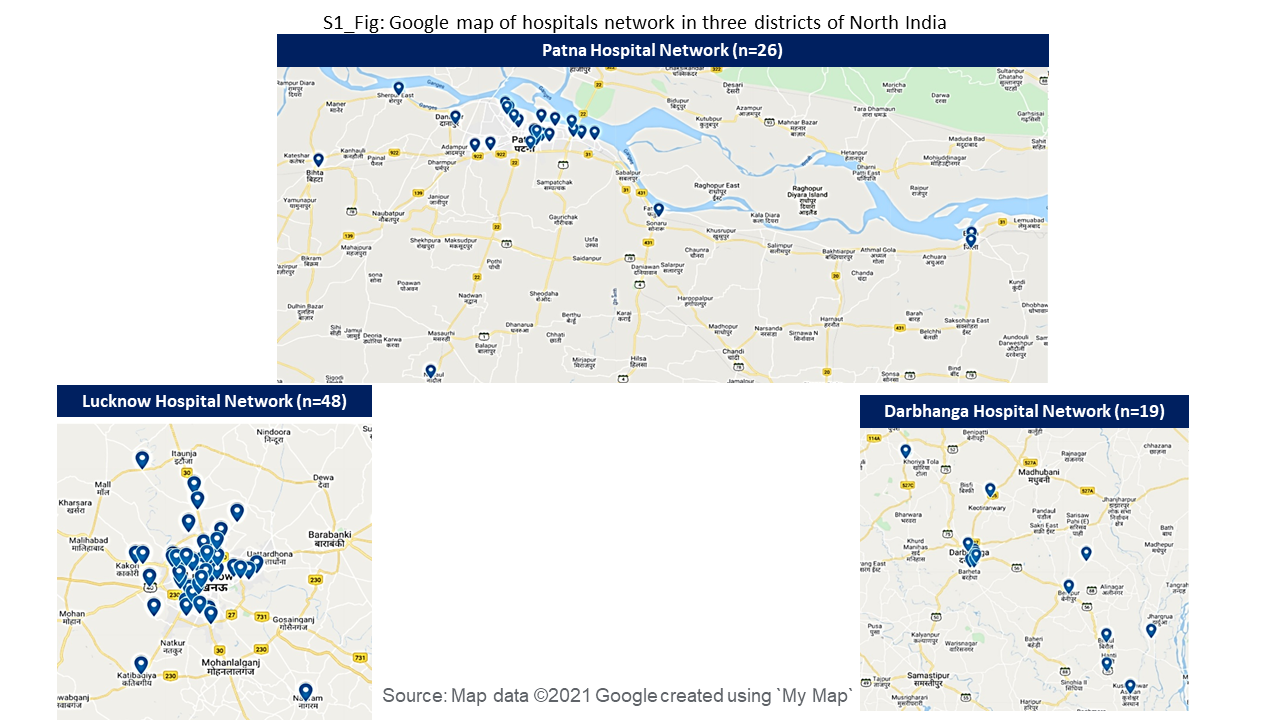

Supplement: S1 Fig — (TIF) [file pone.0276911.s001.TIF]
